# Development of real-time and lateral flow strip reverse transcription recombinase polymerase Amplification assays for rapid detection of peste des petits ruminants virus

**DOI:** 10.1186/s12985-017-0688-6

**Published:** 2017-02-07

**Authors:** Yang Yang, Xiaodong Qin, Yiming Song, Wei Zhang, Gaowei Hu, Yongxi Dou, Yanmin Li, Zhidong Zhang

**Affiliations:** State Key Laboratory of Veterinary Etiological Biology, Lanzhou Veterinary Research Institute, Chinese Academy of Agriculture Sciences, Xujiaping 1, Lanzhou, 730046 Gansu China

**Keywords:** Reverse transcription recombinase polymerase amplification assay, RT-RPA, Lateral flow strip, PPRV, Small ruminants

## Abstract

**Background:**

Peste des petits ruminants (PPR) is an economically important, Office International des Epizooties (OIE) notifiable, transboundary viral disease of small ruminants such as sheep and goat. PPR virus (PPRV), a negative-sense single-stranded RNA virus, is the causal agent of PPR. Therefore, sensitive, specific and rapid diagnostic assay for the detection of PPRV are necessary to accurately and promptly diagnose suspected case of PPR.

**Methods:**

In this study, reverse transcription recombinase polymerase amplification assays using real-time fluorescent detection (real-time RT-RPA assay) and lateral flow strip detection (LFS RT-RPA assay) were developed targeting the N gene of PPRV.

**Results:**

The sensitivity of the developed real-time RT-RPA assay was as low as 100 copies per reaction within 7 min at 40 °C with 95% reliability; while the sensitivity of the developed LFS RT-RPA assay was as low as 150 copies per reaction at 39 °C in less than 25 min. In both assays, there were no cross-reactions with sheep and goat pox viruses, foot-and-mouth disease virus and Orf virus.

**Conclusions:**

These features make RPA assay promising candidates either in field use or as a point of care diagnostic technique.

**Electronic supplementary material:**

The online version of this article (doi:10.1186/s12985-017-0688-6) contains supplementary material, which is available to authorized users.

## Background

Peste des petits ruminants (PPR) is one of the most contagious diseases of small ruminants, which remains endemic in most of Africa, the Middle East, South Asia and China [1–3]. With morbidity and mortality rates as high as 90%, PPR has a devastating impact on the livelihood of the poor and marginal farmers in endemic countries. After the successful global rinderpest eradication program in cattle, PPR has emerged as one of the priority animal diseases whose global control and eradication for poverty alleviation should be considered. To achieve this goal, rapid and accurate diagnosis of PPR would be of important and highly demanded.

The causative agent, PPR virus (PPRV) is a representative member of the genus Morbillivirus of the family *Paramyxoviridae.* PPRV has a genome length of 15,948 nucleotides, which contains six transcriptional units that encode six contiguous and non-overlapping proteins [4]. Genes in PPRV are arranged in an order of 3′-N-P/C/V-M-F-HN-L-5′ [5] and each gene is separated by an intergenic region of variable lengths [6]. Like other negative stranded RNA viruses, the genomic RNA is packaged by nucleoprotein (N) to form nucleocapsid along with phosphoprotein (P) and large protein (L). N protein is a major viral protein produced in highest amount in Morbilliviruses. Based on the N gene, PPRV isolates can be divided into 4 distinct lineages, i.e. lineages I and II only occur in Africa, lineages III occur in Africa and Middle East, and the fourth exists in both Africa and Asia [1, 5]. N protein has been also extensively used in the development of assays for diagnosis of PPR.

Based on clinical signs, PPR may be confused with other diseases, such as capripox, foot-and-mouth disease and Orf, which also affect small ruminants. Therefore, laboratory tests must be performed for a definitive diagnosis of PPR. Different cell lines have been used to isolate PPRV but with limited success [7, 8]. Thus, an ELISA for detection of viral nucleoprotein was developed as an alternative method to virus isolation [9]. However, these methods are laborious and time-consuming, and may not be used as pen-side tests. Recently, an immunochromatographic assay was developed for rapid detection of PPRV, however, its sensitivity was not good enough to detect PPRV in clinical samples with a low virus load [10]. Detection of serum antibody is also not effective because all assays based on detection of PPRV antibody could not differentiate infected animals from vaccinated animals [11–13]. As a result of its rapid, accurate, and sensitive nature, conventional reverse transcription polymerase chain reactions (RT-PCRs) are widely used for detection of PPRV genomic material [14, 15]. However, these conventional RT-PCR assays also have shortcomings, such as being labor intensive, time consuming and high risk of cross-contamination [16]. Real time RT-PCR assay has acquired extensive adoption over conventional RT-PCR and been developed for detection and quantitation of PPRV present in clinical samples [16–20]. But the real time RT-PCR assay still relies on specialized and expensive thermos cycling machine, as a result it is difficult to be used as a “pen-side” test and in endemic areas with low resources. Loop-mediated isothermal amplification (LAMP) provides an isothermal method to amplify viral RNA without requirement of a specific thermal cycler. The LAMP reaction could be performed using a water bath, and the reaction results could be directly distinguished through color change by the naked eye. LAMP could adapt into different formats for detection of amplified products, such as agarose gel, lateral flow analysis (visual monitoring) and real-time fluorescence analysis [21, 22]. However, RT-LAMP assay requires six primers and has unsatisfactory reliability in detection of highly variable viruses [21]. In recent years, a recombinase polymerase amplification (RPA) assay was developed for rapid detection of different viruses of veterinary importance, such as foot-and-mouth disease virus (FMDV) [23], Orf virus (ORFV) [24], Schmallenberg virus (SBV) and bovine viral diarrhea virus (BVDV) [21]. These studies had shown that RPA assay was characterized with high sensitivity, good diagnostic specificity and rapidity, and represents a powerful potential to be a pen-side assay for rapid detection of pathogens. The amplification of RPA relies on a specific combination of recombinase, single strand binding protein, and strand displacing DNA polymerase. Twist Amp® exo probe is needed in real-time fluorescent probe-based RPA assay, in which generation of fluorescence signal relies on the detaching off fluorophore and quencher at an internal abasic site mimic (tetrahydrofuran, THF) of the Twist Amp® exo-probe using DNA repair enzyme Exonuclease III. The real-time fluorescence signal is measured using fluorescence detection equipment. For lateral-flow strip (LFS) based RPA assay, Twist Amp® nfo probe is required. In the present study, reverse transcription RPA assays with real-time fluorescent detection (real-time RT-RPA assay) and lateral-flow strip detection (LFS RT-RPA assay) were developed based on the N gene of PPRV. The results showed that both PPRV real-time and LFS RT-RPA assays developed are sensitive and specific for detection of PPRV.

## Methods

### Virus strains

All viruses used in this study were preserved in our laboratory: peste des petits ruminants virus (PPRV) Nigeria 75/1; Orf virus (ORFV)/Vaccine/CHA; goat pox viruses (GPV) AV40, sheep pox viruses (SPV) Gulang2009; foot-and-mouth disease virus (FMDV)/O/CHA; FMDV/A/CHA and FMDV/Asia I/CHA.

### Sample preparation

To prepare PPRV-spiked tissues lysates, PPRV-free tissues samples of liver, lungs, stomach, kidney, lymphatic nodes and skin (*n* = 18, three each tissue) were collected from three healthy sheep. 10% (w/v) tissue suspensions (one milligram tissue samples were homogenized with nine volumes PBS using MP FastPrep-24) were then prepared by homogenizing tissue samples in PBS. Following a brief centrifugation, the homogenized tissue samples were spiked with 10^4^ TCID_50_ of PPRV Nigeria 75/1 and stored at -80 °C until used. Known PPRV positive samples (*n* = 14) and known negative samples (*n* = 10) stored in our laboratory were confirmed using a real time RT-qPCR assay [19]. Thirty-two clinical samples collected from suspected cases of PPR in Gansu Province, China and five samples obtained from healthy sheep, which were screened with the RT-qPCR assay to confirm the presence or absence of PPRV-RNA, were also tested using the newly developed RT-RPA assay.

### Viral RNA extraction

Total RNA was extracted from samples using High Pure Viral Nucleic Acid kit (Roche) according to the manufacturer’s instructions and eluted in a final volume of 40 μL. Extracted RNA was stored at -80 °C until further use for all assays in this study.

### Generation of RNA standard

The PPRV N gene segments (354 bp, ranging from 528 bp to 881 bp of X74443) were synthesized by Genewiz (Suzhou, China) and cloned into a pUC57 vector downstream of the T7 promoter, designated as pPPRV/RPA. The pPPRV/RPA plasmid was extracted using Plasmid Mini kit I (Promega, USA) and the plasmid was linearized downstream of the PPRV N gene segments. A total of 1 μg of linearized product was used for in vitro transcription (Megascript® kit, Ambion, USA) following the manufacturer’s instructions. Transcribed RNA was DNase treated and purified using an RNA clean-up protocol (RNeasy Mini KIT, Qiagen, Germany) according to the manufacturer’s instructions. The purified RNA was quantified by Nanovue (GE lifescience, USA) and stored at -80 °C until used.

### Primers and probe design and optimizing for RPA

PPRV-specific RPA primers and probe, based on the N gene of PPRV, were designed according to RPA guidelines (Twist Amp® DNA amplification kits combined instruction manual) from TwistDx (Cambridge, United Kingdom). Primers and probe were aligned with the N gene consensus sequence of X74443 (LII, Nigeria), L39878 (LII, Nigeria), EU267274 (LII, Nigeria), EU267273 (LI, Cote d’Ivoire), AJ849636 (LIV, Turkey), FJ905304 (LIV, China Tibet), AY560591 (LIV, India Sungri), AJ563705 (LIV, Turkey), KJ867543 (LIII, Uganda) and KJ867544 (LIII, Oman). All PPRV N gene sequences above were retrieved from GenBank and multiple sequence alignment of the gene sequences were manually designed based on the PPRV N gene as recommendation by TwistDx (Cambridge, UK). All primers and probes were synthesized by Sangon Biotech (Shanghai, China). The sequences of all the primers and probe were shown in Table 1. In order to determine the most efficient primer pair for PPRV real-time RT-RPA assay, three forward primers (Fe1 N to Fe3 N) and reverse primers (Re1 N to Re3 N) targeting different regions of N gene were designed based on the alignment of all available sequences of PPRV N gene (Table 1). Nine different combinations of primers (i.e. Fe1 N/Re1 N; Fe1 N/Re2 N; Fe1 N/Re3 N; Fe2 N/Re1 N; Fe2 N/Re2 N; Fe2 N/Re3 N; Fe3 N/Re1 N; Fe3 N/Re2 N; Fe3 N/Re3 N) were then tested with the probe (Pe) in the PPRV real-time RT-RPA assay.

**Table 1:** RPA primers and probes designed in this study

Name	Sequence (5′–3′)	Genome location(X74443)
PPRV-RPA Fe1 N	GAAGAGTTCAATATGTTGTTAGCCTCCAT	588–616
PPRV-RPA Fe2 N	CCAAGGCGGTTACGGCACCGGATACGGCAGCTGAC	643–677
PPRV-RPA Fe3 N	TTACGGCACCGGATACGGCAGCTGACTCAGAACTG	652–686
PPRV-RPA Re1 N	TTTGTCAAGGCGAAATTCCCCAATCACTCTCC	715–746
PPRV-RPA Re2 N	ACTGCGTCCAGCCACCCTTTGTCAAGGCGAAATTC	729–763
PPRV-RPA Re3 N	ACTGCGTCCAGCCACCCTTTGTCAAGGCGAAA	732–763
PPRV-RPA Pe	GATACGGCAGCTGACTCAGAACTGAGAAGG (FAM-dT)G(THF)G(BHQ1-dT)TAAATACACACAACA - C3 space	663–712
PPRV-RPA Fn1 N	GAAGAGTTCAATATGTTGTTAGCCTCCAT	588–616
PPRV-RPA Fn2 N	CCAAGGCGGTTACGGCACCGGATACGGCAGCTGAC	643–677
PPRV-RPA Fn3 N	TTACGGCACCGGATACGGCAGCTGACTCAGAACTG	652–686
PPRV-RPA Rn1 N	Biotin-TTTGTCAAGGCGAAATTCCCCAATCACTCTCC	715–746
PPRV-RPA Rn2 N	Biotin-ACTGCGTCCAGCCACCCTTTGTCAAGGCGAAATTC	729–763
PPRV-RPA Rn3 N	Biotin-ACTGCGTCCAGCCACCCTTTGTCAAGGCGAAA	732–763
PPRV-RPA Pn	FAM-TACGGCAGCTGACTCAGAACTGAGAAGGTG	663–712
	-THF-GTTAAATACACACAA-C3 space	

PPRV-RPA F and R, RPA primer; PPRV-RPA P, RPA Exo probe; BHQ1-dT, thymidine nucleotide carrying Black Hole Quencher 1; THF, tetrahydrofuran spacer; FAM-dT, thymidine nucleotide carrying fluorescein; C3 space, block elongation; “F”, “R”, “N”, “P”, “e” and “n” were defined as forward primer, reverse primer, nucleoprotein, probe, RPA exo kit and RPA nfo kit, respectively

### Real-time RT-qPCR assay

Real-time RT-qPCR assay was performed in Agilent Technologies Stratagene Mx3005P thermocycler (Life technologies, USA) using the Superscript III/Platinum Taq One-step RT-qPCR kit (Invitrogen, USA) [19]. The reactions were prepared as a 25 μL reaction volume containing 2 μL extracted RNA, 12.5 μL Superscript III/Platinum Taq One-step RT-qPCR reaction mix, 1 μL Superscript III/Platinum Taq One-step RT-qPCR enzyme mix, 0.5 μL ROX reference dye as a passive reference, 5 pmol Taqman probe, and 10 pmol of forward and reverse primers. The following thermal profile was used: an initial reverse transcription at 45 °C for 30 min, followed at 95 °C for 10 min and 40 cycles of amplification (15 s at 94 °C and 1 min at 60 °C). The data were analyzed using Mx3005P System software.

### Real-time RT-RPA assay

Real-time RT-RPA assay was performed in a 50 μL volume using the Twist Amp® exo RT kit (TwistDx, Cambridge, United Kingdom), which consisted of 29.5 μL rehydration buffer, 2 μL template RNA, 2.1 μL of forward and reverse primers (10 μM, Beijing Genomics Institute, China), 0.3 μL of RPA exo probe (10 μM, Sangon Biotech, China), 11.8 μL of ddH_2_O and 2.5 μL of magnesium acetate (280 mM). Optimal reaction conditions were defined after testing different incubation temperatures (39 to 42 °C), as well as different concentrations of template (0.5 μL to 2 μL) and magnesium acetate (1 μL to 2.5 μL). Fluorescence measurements using the FAM channel were performed for 20 min at an optimized temperature (40 °C). Fluorescence intensity of FAM was determined every 20 s. A sample was deemed positive if all replicates were three and a half standard deviations (3.5 SD) above the background during a defined time range (i.e. after 19 to 20 min of amplification). A threshold time range of 0 to 4 min and 30 s was used.

### Lateral-flow strip RT-RPA assay

Lateral-flow strip RT-RPA assay (LFS RT-RPA assay) was performed using the Twist Amp® nfo kit from TwistDx (Cambridge, United Kingdom) according to the manufacturer’s instruction. Briefly, the reverse primer with a 5′-biotin label was used. The probe consists of an upstream stretch (30 bp) carrying a 5′-FAM antigenic label, which is connected via a THF spacer to an adjacent downstream oligonucleotide (15 bp) carrying a C3-spacer (polymerase extension blocking group) at its 3′end. All oligonucleotides in this study were synthesized by Sangon Biotech (Shanghai, China). The extracted PPRV RNA was first reverse transcribed into cDNA before RPA reaction was carried out with the Twist Amp® nfo kit, as the kit did not contain reverse transcriptase. Synthesis of cDNA was carried out in 20 μL reaction using transcriptor first Strand cDNA Synthesis kit (Roche, Germany) following manufacturer’s instructions. Briefly, 1 μL anchored-oligo primer was added to 12 μL of the purified RNA. After incubation at 65 °C for 10 min, the mixture was immediately placed on ice for 2 min and a mixture containing 4 μL RT buffer (5×), 0.5 μL Protector RNase inhibitor, 2 μL Deoxynucleotide Mix and 0.5 μL transcriptor reverse transcriptase were added. The reaction was carried out at 50 °C for 60 min and 85 °C for 5 min. PPRV RPA nfo reactions were then performed in a 50 μL volume with the Twist Amp® nfo kit (TwistDx, UK) containing 420 nM nfo RPA primers, 15 nM RPA probes, 2.5 μL of 280 mM MgAc, 2 μL of cDNA template and 1× rehydration buffer. The RPA reaction was carried out in the heating block at optimized temperature (39 °C) for 20 min, and then Hybridetect 2 T lateral flow stripe (LFS) (Milenia Biotec GmbH, Germany) dipsticks were used to detect amplicons. One μL of the amplified product was diluted in 99 μL of the assay buffer (Tris-buffered saline). The strips were then placed into the mixture and incubated at an upright position. The final result was read visually after incubation for 2 min at room temperature. A testing sample was considered positive when both the detection line (sample testing line) and the control line (anti-rabbit antibody line) were visible. A testing sample was considered negative when only the control line was visible. The amplicons could also been analyzed on 2% agarose-gel electrophoresis (Agarose Broad Range, Germany) stained with ethidium bromide after being purified with PCR clean-up Kit (Qiagen, Germany) to further confirm the testing result.

### Sensitivity and specificity of PPRV real time RT-RPA and LFS RT-RPA assays

To test the dynamic range of PPRV real-time RT-RPA assay, the standard RNA in vitro transcribed from the pPPRV/RPA plasmid was diluted 10-fold using ddH_2_O, ranging from 10^6^ to 10^1^ genome copies per reaction and then tested by PPRV real-time RT-RPA assay. Every run was repeated six times. The sensitivity of PPRV real-time RT-RPA assay was further evaluated with spiked samples as described above. The sensitivity of PPRV LFS RT-RPA assay was determined by running 10-fold serial dilutions (10^6^ to 10^1^ genome copies per reaction) of the standard RNA. Additionally, the amplified products in the PPRV LFS RT-RPA reaction was also tested by subsequent visualization with agarose gel electrophoresis. The specificity of both PPRV real-time RT-RPA and LFS RT-RPA assays were evaluated with FMDV serotypes O, A and Asia 1, ORFV, GPV and SPV, which caused similar clinical signs to PPR in small ruminants.

### Statistics analysis

The semi-log regression analyses of the sensitivity of PPRV real time RT-RPA assay were performed using PRISM 5.0 software (GraphPad Software, USA), and the probit analysis were performed by Statistica software (StatSoft, Hamburg, Germany). Comparison between values of real-time RT-RPA threshold time and values of real-time RT-qPCR cycle threshold for PPRV detection was performed by linear regression analysis using Excel software.

## Results

### Sensitivity and specificity of PPRV real-time RT-RPA assay

**Fig. 1:**
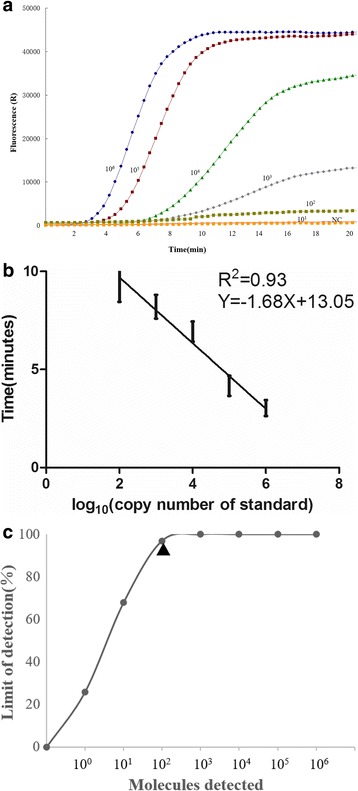
**a** Typical raw fluorescence data of real-time RT-RPA assay for standard RNA in vitro transcribed from the pPPRV/RPA plasmid as they are shown in the figure. NC represent negative control. **b** Reproducibility of the real-time RT-RPA assay. The threshold time is represented as mean ± standard deviation (SD). The standard regression line was generated based on 6 data sets (**c**) Probit regression analysis using Statistics software (GraphPad Prism 5) was done on data from the six runs of real-time RT-RPA assay. The limit of detection at 95% probability is depicted by a triangle

The RT-RPA assay result showed that the primer set Fe2 N/Re2 N yielded the highest efficiency of amplification (Additional file 1: Figure S1). Therefore, this pair of primers was employed in PPRV RT-RPA assay for further validation. As shown in Fig. 1a, the dynamic detection range of the assay spans 5 logs ranging from 6 to 2 log copies per reaction, with the corresponding threshold time ranging from 3 min at 10^6^ copies per reaction to 7 min at 10^2^ copies per reaction at 40 °C. This result indicates that there is a wide dynamic range for quantifying target RNA in PPRV real-time RT-RPA assay (Fig. 1a and b). The detection limit of PPRV real-time RT-RPA assay at 95% probability was 10^2^ copies per reaction (probit analysis, *p* ≤ 0.05) (Fig. 1c). The specificity of PPRV real-time RT-RPA assay was determined by cross detection of other viruses which infect epithelium or mucus in sheep and goat, including FMDV serotypes O, A and Asia 1, ORFV and capripox virus. No cross detection was observed, indicating the assay was specific for PPRV (Table 2).

**Table 2:** Comparison of PPRV real-time RT-RPA assay and PPRV LFS RT-RPA assay with RT-qPCR assay on virus species, spiked samples, field samples and healthy sheep samples

Sample type	Sample name	real-time RT-RPA(min)	LFS RT-RPA)	RT-qPCR (Cq	specific RT-qPCR(Cq)
Virus species	PPRV Nigeria 75/1	4.6	+	18	18
	GPV AV40	-	-	-	17
	SPV Gulang2009	-	-	-	19
	ORFV/Vaccine/CHA	-	-	-	20
	ORFV/HB/CHA	-	-	-	22
	FMDV/O/CHA	-	-	-	21
	FMDV/A/CHA	-	-	-	19
	FMDV/Asia1/CHA	-	-	-	20
Spiked samples	Liver 1	4.6	+	16	-
	Liver 2	7.6	+	28	-
	Liver 3	6	+	20	-
	Lungs 1	4.3	+	15	
	Lungs 2	5.3	+	17	-
	Lungs 3	6	+	21	-
	Stomach 1	6	+	23	-
	Stomach 2	4	+	16	-
	Stomach 3	5.3	+	18	-
	Kidney 1	5.6	+	21	-
	Kidney 2	6	+	24	-
	Kidney 3	3.6	+	14	-
	Lymphatic nodes 1	5.3	+	17	-
	Lymphatic nodes 2	6	+	21	-
	Lymphatic nodes 3	4	+	18	-
	Skin 1	5.6	+	22	-
	Skin 2	6	+	25	-
	Skin 3	7.6	+	27	-
Field samples	Lung	-	-	-	-
	Spleen	-	-	-	-
	Nasal swab	-	**-**	-	-
	Kidney	-	**-**	-	-
	Spleen	6.3	+	25	-
	Nasal swab	-	-	-	-
	Nasal swab	-		-	-
	Kidney	-	-	31	-
	lymphatic nodes	-	-	-	-
	Nasal swab	6	+	23	-
	Kidney	-	-	-	-
	Spleen	4	+	19	-
	Nasal swab	-	-	-	-
	Lung	5	+	19	-
	Spleen	-	-	-	-
	Kidney	-	-	33	-
	Spleen	-	-	-	-
	Lung	-	-	-	-
	Kidney	5	+	19	-
	Nasal swab	7	+	24	-
	Spleen	6.3	+	20	-
	Lung	6.6	+	22	-
	Spleen	5.3	+	20	-
	Kidney	6	+	20	-
	Spleen	5	+	18	-
	Nasal swab	7.3	+	26	-
	Kidney	7.6	+	27	-
	Spleen	5.6	+	21	-
	Lung	6.3	+	22	-
	Lymphatic nodes	7.3	+	27	-
	Kidney	6.6	+	24	-
	Spleen	6	+	23	-
Healthy sheep samples	Liver	-	-	-	-
	Lungs	-	-	-	-
	Stomach	-	-	-	-
	Kidney	-	-	-	-
	Skin	-	-	-	-

Samples include PPRV、sheep and goat pox viruses 、foot-and-mouth disease virus and Orf virus, eighteen spiked samples, eighteen filed samples and five healthy sheep samples. *CaPV* capripoxvirus, *GPV* goat pox viruses, *SPV* sheep pox viruses, *CHA* China. *specific qPCR* specific qPCR tested the respective viruses used in this study, + : positive; - : negative

**Fig. 2:**
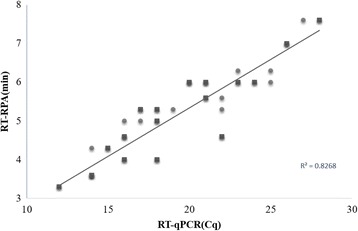
Comparison between performances of PPRV real-time RT-RPA assay and real-time RT-PCR assay on positive samples (*n* = 14, marked by balls) and samples of spiked tissues lysates (*n* = 18, marked by diamonds). Linear regression analysis of the real-time RT-RPA threshold time (y axis) and RT-qPCR cycle threshold (Cq) values (x axis) were determined by Excel software

The developed PPRV real-time RT-RPA assay was then further tested using samples of PPRV-spiked tissue lysates (*n* = 18). Results showed that all samples of the virus-spiked tissues were tested as positive for PPRV. There were no amplicons detected in the non-virus-spiked samples despite threshold time being over 30 min. These results consisted with the results of PPRV real-time RT-qPCR assay. Both assays showed the same performance on the samples above, and a correlation was found between values of the cycle threshold (RT-qPCR) and threshold time (RT-RPA) (R squared 0.83, Fig. 2).

### Sensitivity and specificity of PPRV LFS RT-RPA assay

**Fig. 3:**
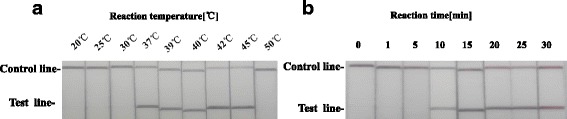
Determination of reaction temperature and time. **a** The LFS RT-RPA assay were performed respectively at different temperatures as they are shown in the figure. **b** After 10 min of isothermal amplification reaction, the slight test line is visible on the test strip, and when the amplification time is longer than 15 min or 20 min, the clear test line is visible on the test strip. Including the incubation time of 2 min, the whole assay time of the LFS RT-RPA assay is less than 25 min, and the results showed that this assay works effectively in a broad range of temperatures from 37 to 45 °C

**Fig. 4:**
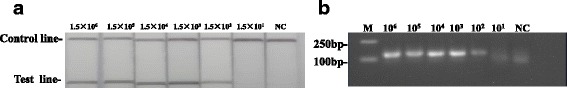
**a** The sensitivity of LFS RT-RPA was 150 copies of the standard plasmids transcribed RNA, **b** and all the LFS RT-RPA positive result could be consolidated on stained agarose gel (2%) assay

**Fig 5:**
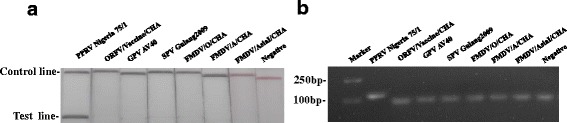
Specificity test results of LFS RT-RPA assay using total DNA/RNA extracted from PPR virus and other virus. PPRV: peste des petits ruminants, ORFV: Orf virus, GPV: goat pox viruses, SPV: sheep pox viruses, FMDV: foot-and-mouth disease virus. **a** Positive RPA nfo reaction products can be detect in the lateral flow strips format (LFS RT-RPA). **b** Positive RPA nfo reaction products (121 bp) also can be detect on a stained agarose gel (2%)

To determine the optimal temperature for the RT-RPA reaction, the ability of the PPRV LFS-RT-RPA assay was tested to amplify 10^4^ copies of PPRV RNA as template at a range of temperatures from 20 °C to 50 °C. Initially, we assessed this range of temperature under incubation time of 30 min. As shown in Fig. 3a, no amplification products were observed in reactions incubated at <37 °C and at 50 °C. There were no differences in amplification at 37, 39, 40, 42 and 45 °C (Fig. 3a). Thus, 39 °C was selected arbitrarily as the PPRV LFS RT-RPA assay standard temperature. The performance of the PPRV LFS-RT-RPA assay was tested at 39 °C incubated for 0, 1, 5, 10, 15, 20, 25 and 30 min. As shown in Fig. 3b, no amplified products were observed in reactions incubated for less than 10 min and weak amplified product observed for 10 min. When incubation time increased from 15 to 30 min, the assay performance was improved, and there were no differences in amplification in reactions incubated between 15 and 30 min, thus 20 min was selected arbitrarily as the standard incubation time for PPRV LFS RT-RPA assay. The detection limit of the PPRV LFS RT-RPA assay was determined using a dilution series of the cDNA (corresponding to 10^6^ to10^1^ RNA copies per reaction). The test results showed that the PPRV LFS RT-RPA assay gave clear positive signal at a concentration of 150 copies per reaction (Fig. 4a), and agarose gel electrophoresis confirmed the results (Fig. 4b). In evaluating the specificity of PPRV LFS RT-RPA assay, no cross reactions were observed with FMDV serotypes O, A and Asia 1, ORFV, SPV and GPV (Fig. 5a), which was confirmed by the agarose gel electrophoresis (Fig. 5b). The specificity of the PPRV LFS RT-RPA assay was further validated with known positive samples (*n* = 14) and negative samples (*n* = 10) which was confirmed by PPRV real-time RT-qPCR assay. It was found that PPRV LFS RT-RPA assay was able to correctly identify all samples examined. These results indicated that the PPRV LFS RT-RPA assay were specific for detection of PPRV.

### Performance of PPRV real-time RT-RPA and LFS RT-RPA assays on clinical samples

Both PPRV real-time RT-RPA and LFS RT-RPA assays were further evaluated with clinical samples including thirty-two samples collected from suspected cases of PPR and five samples from healthy sheep. The results were then compared with PPRV real-time RT-qPCR assay. No amplification was detected in five samples obtained from healthy sheep with both PPRV real-time and LFS RT-RPA assays, even though threshold time (min) exceeded 30 min. Of 32 samples collected from suspected cases of the PPR, 20 samples were found to be positive by real-time PPRV RT-qPCR assay (CT value ranging from 16 to 33). Among these samples, 18 samples were determined to be positive by both PPRV real-time RT-RPA assay (threshold time ranging from 5 to 7.6 min) and PPRV LFS RT-RPA assay. The detection limit of PPRV real-time RT-qPCR assay was 10 copies per reaction, corresponding to a mean CT value of 34.08, while the detection limit of developed LFS RT-RPA assay was 150 copies per reaction, corresponding to a mean CT value of 30.76. For the two samples tested as negative by both PPRV real-time RT-RPA and LFS RT-RPA assays, the CT values of RT-qPCR assay values were 33 and 31, respectively, which were beyond the detection limit of PPRV LFS RT-RPA assay. Based on a total of 37 clinical samples examined, the sensitivity and the specificity of PPRV real-time RT-RPA assay and LFS RT-RPA assay for identification of PPRV was 90 and 100%, respectively, when compared to PPRV real-time RT-qPCR (Tables 2 and 3).

**Table 3:** Performance of the real-time RT-RPA assay or LFS RT-RPA assay in comparison with the real-time RT-qPCR assay for detecting PPRV on clinical samples

		Real-time qPCR		Performance characteristics (%)	
		Positive	Negative	Sensitivity	Specificity
Real-time RPA	Positive	18	0	90%	100%
	Negative	2	17		
	Total (*n* = 37)	20	17		
RPA LFD	Positive	18	0	90%	100%
	Negative	2	17		
	Total (*n* = 37)	20	17		

Samples include thirty-two samples collected from suspected cases of the sheep, and five serum samples collected from healthy sheep

## Discussion

The developed PPRV real-time RT-RPA and LFS RT-RPA assays using F2 N/R2 N primer set based on PPRV N gene sequences could sensitively and specifically detect PPRV present in samples. It is worth mentioning that maximal running time for the developed RPA assays is less than 25 min, regardless of the viral concentration in samples as long as it is above the detection limit (i.e. > 100 copies per reaction). The results in this study demonstrated that both developed PPRV real-time RT-RPA and LFS RT-RPA assays are highly specific for detection of PPRV as there is no cross reaction with GPV, SPV, FMDV and ORFV, which may cause similar clinical signs to PPRV in small ruminants, indicating the potential possibility of being a novel testing tool for differential diagnosis. Its high specificity for detection of PPRV is due to the fact that RPA uses specific detection probes, like real-time qPCR assay does. Although the sensitivity of RPA is lower than RT-qPCR, some advantages of the RT-RPA assay over RT-qPCR assay make it attractive. Firstly, reaction mixtures are pre-made pellets and provided in vacuum-sealed pouches, which can be kept at room temperatures for several days. This would save on cooling costs and facilitate onsite diagnosis of PPR in the field. Secondly, the reaction can be performed in a water bath at temperature of 37 °C to 45 °C for 20 min. Thirdly, RPA supplies different kinds of end-point detection formats (such as Twist Amp® basic kit, Twist Amp® exo kit and Twist Amp® nfo kit) that could be adapted by different laboratories, therefore making RPA adaptable in a well-equipped laboratory, a mobile laboratory or a rural area where expensive diagnostic instrument is not available.

Like RT-LAMP assay, RT-RPA reaction could also be performed using a water bath and the reaction results could be directly distinguished through LFS device by the naked eye. However, the developed RT-RPA assay was faster than RT-LAMP assay (within 20 min in RT-RPA assay while 60 min in RT-LAMP assay). Moreover, RT-RPA assay requires one pair of primers combined with a probe and lower temperature (39 °C), while RT-LAMP needs at least three pairs of primers and higher temperature (62 °C). However, as a relatively novel technology RPA has not been used in field diagnostics yet. Like other molecular assays including LAMP [22, 25, 26], RNA used in the RPA assay has been extracted from samples which are laborious and time-consuming. To achieve as a pen-site molecular diagnostic tool, there is a need to simplify RNA or DNA extraction. We have successfully used a commercially available innuPREP MP basic kit A (Jena Analytik, Jena, Germany) and a magnetic bead separation rack combined with proteinase K to extract nucleic acids from all samples. The extracted nucleic acids could be directly used in the PCR assay and RPA assay. Our test results with the 32 clinical samples, as described above, using the innuPREP MP basic kit A showed the same performance in both RT-RPA assay and RT-qPCR assay when compared with the High Pure Viral Nucleic Acid kit (Roche, Germany). At the moment, PPRV RPA assay is still costlier than LAMP assay and RT-qPCR assay, but RPA has a few unique advantages as described above. As the technology develops and becomes widely used for diagnosis of disease, the cost of RPA should gradually drop. In fact, we have successfully reduced the cost by simple reduction of reaction volume from the original 50 μL volume to 25 μL.

## Conclusions

PPRV real-time RT-RPA and LFS RT-RPA assays described are sensitive and specific for rapid detection of PPRV within less than 20 min. Evaluation using extracted RNA from clinical samples demonstrated both assay have about 90% sensitivity and 100% specificity when compared to PPRV real-time RT-qPCR assay. The results are encouraging but the assays need to be further validated by analysis of a larger number of samples from animals infected with PPRV and with isolates of different PPRV lineages II, III, IV before such an assay can be considered for routine diagnostic use.

## Additional file

Additional file 1: Figure S1. Three forward primes (Fe1 N to Fe3 N), three reverse primers (Re1 N to Re3 N) and one probe (Pe) were tried to screen the best combination yielding the highest amplification. The amplification results of nine different combinations were shown in the figure. (PPTX 126 kb)

## Abbreviations

LFS RT-RPA assay: Reverse transcription recombinase polymerase amplification assay combination with a lateral flow strip
PPRV: Peste des petits ruminants virus
real-time RT-RPA assay: Reverse transcription recombinase polymerase amplification assay using real-time fluorescent detection
RPA: Recombinase polymerase amplification
RT-LAMP: Reverse transcription loop-mediated isothermal amplification
RT-qPCR: Reverse transcription quantitative PCR
