# Supplementary material for: Development of real-time and lateral flow strip reverse transcription recombinase polymerase Amplification assays for rapid detection of peste des petits ruminants virus
Source: Virol J. 2017 Feb 7;14:24. doi: 10.1186/s12985-017-0688-6 (PMC5297045; doi:10.1186/s12985-017-0688-6)
Supplement: Additional file 1: Figure S1. — Three forward primes (Fe1 N to Fe3 N), three reverse primers (Re1 N to Re3 N) and one probe (Pe) were tried to screen the best combination yielding the highest amplification. The amplification results of nine different combinations were shown in the figure. (PPTX 126 kb) [file 12985_2017_688_MOESM1_ESM.pptx]

## Slide 1
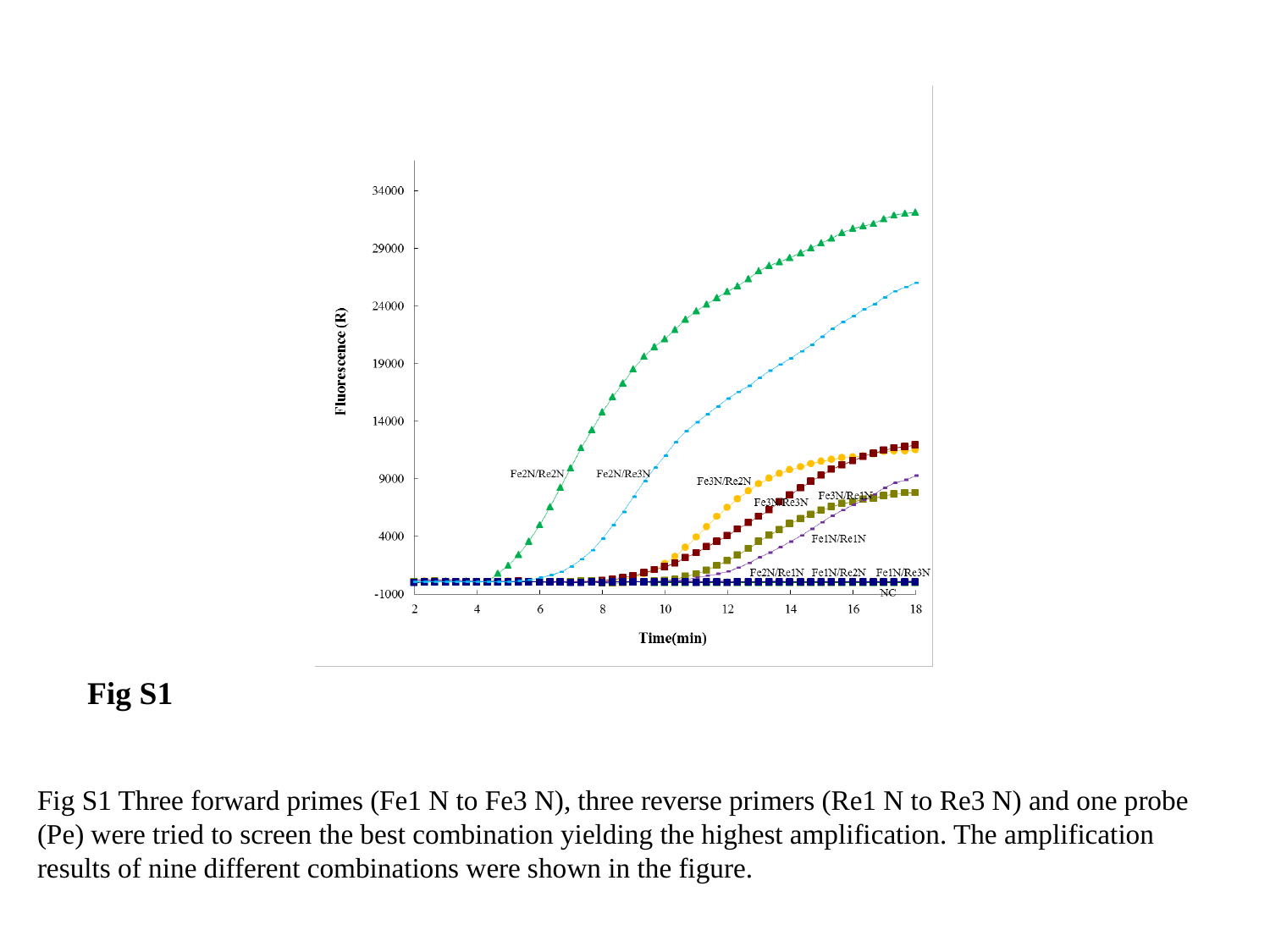

Fig S1
Fig S1 Three forward primes (Fe1 N to Fe3 N), three reverse primers (Re1 N to Re3 N) and one probe (Pe) were tried to screen the best combination yielding the highest amplification. The amplification results of nine different combinations were shown in the figure.
